# Interplay between Genetic and Clinical Variables Affecting Platelet Reactivity and Cardiac Adverse Events in Patients Undergoing Percutaneous Coronary Intervention

**DOI:** 10.1371/journal.pone.0102701

**Published:** 2014-07-22

**Authors:** Jolanta M. Siller-Matula, Irene M. Lang, Thomas Neunteufl, Marek Kozinski, Gerald Maurer, Katarzyna Linkowska, Tomasz Grzybowski, Jacek Kubica, Bernd Jilma

**Affiliations:** 1 Department of Cardiology, Medical University of Vienna, Vienna, Austria; 2 Department of Cardiology and Internal Medicine, Collegium Medicum of the Nicolaus Copernicus University, Bydgoszcz, Poland; 3 Institute of Molecular and Forensic Genetics, Collegium Medicum of the Nicolaus Copernicus University, Bydgoszcz, Poland; 4 Department of Clinical Pharmacology, Medical University of Vienna, Vienna, Austria; King's College London School of Medicine, United Kingdom

## Abstract

Several clinical and genetic variables are associated with influencing high on treatment platelet reactivity (HTPR). The aim of the study was to propose a path model explaining a concurrent impact among variables influencing HTPR and ischemic events. In this prospective cohort study polymorphisms of CYP2C19*2, CYP2C19*17, ABCB1, PON1 alleles and platelet function assessed by Multiple Electrode Aggregometry were assessed in 416 patients undergoing percutaneous coronary intervention treated with clopidogrel and aspirin. The rates of major adverse cardiac events (MACE) were recorded during a 12-month follow up. The path model was calculated by a structural equation modelling. Paths from two clinical characteristics (diabetes mellitus and acute coronary syndrome (ACS)) and two genetic variants (CYP2C19*2 and CYP2C19*17) independently predicted HTPR (path coefficients: 0.11 0.10, 0.17, and -0.10, respectively; p<0.05 for all). By use of those four variables a novel score for prediction of HTPR was built: in a factor-weighted model the risk for HTPR was calculated with an OR of 3.8 (95%CI: 3.1–6.8, p<0.001) for a score level of ≥1 compared with a score of <1. While MACE was independently predicted by HTPR and age in the multivariate model (path coefficient: 0.14 and 0.13, respectively; p<0.05), the coexistence of HTPR and age ≥75 years emerged as the strongest predictor of MACE. Our study suggests a pathway, which might explain indirect and direct impact of variables on clinical outcome: ACS, diabetes mellitus, CYP2C19*2 and CYP2C19*17 genetic variants independently predicted HTPR. In turn, age ≥75 years and HTPR were the strongest predictors of MACE.

## Introduction

Within the past years, platelet function studies have shown that up to 50% of clopidogrel treated patients have high on-treatment platelet reactivity (HTPR) [1]. Studies in over 20.000 of patients indicate an up to 10-fold higher risk of adverse ischemic events in patients with HTPR [2]–[9]. Several clinical and genetic variables are associated with HTPR. The clinical factors include obesity, renal dysfunction, diabetes, age, heart failure, inflammation and acute coronary syndromes (ACS) [10]–[13]. From multiple candidate genes being involved in metabolism of clopidogrel, the CYP2C19*2 (loss-of-function allele) and the CYP2C19*17 (gain-of-function mutation), have been associated with response variability to clopidogrel in some studies [12], [14]–[19]. Whether other polymorphisms of genes being involved in the metabolism or action of clopidogrel (e.g. the intestinal efflux transport pump P-glycoprotein pump encoded by the ABCB1 gene or the paraoxonase-1, PON1 gene) predict HTPR is a matter of debate [14], [20]–[23].

To our knowledge, a concurrent impact among different variables influencing HTPR and ischemic events has not been reported. Therefore, the aim of the current paper was to investigate the interaction of clinical and genetic risk factors of HTPR in relation to cardiac ischemic events.

## Methods

### Study design

This paper reports a sub-analysis of a previously published prospective observational cohort study [24]. The study design has been reported in detail [24]. In brief, the Ethics Committee of the Medical University of Vienna approved the study protocol in accordance with the Declaration of Helsinki. Participants were included into the study between March 2007 and September 2008, and followed up for 12 months. Clinical follow up information was obtained by contacting all patients by phone and/or mail every three months, and by queries from the national death registry. Inclusion criteria were: written informed consent obtained before study entry, previous stent implantation, PCI at least 2 h after clopidogrel loading with 600 mg, age >18 years and planned treatment with clopidogrel and aspirin for 12 months. The only exclusion criterion was participation in other interventional trials. Four hundred sixteen patients with coronary artery disease (CAD) undergoing percutaneous coronary intervention (PCI) were consecutively enrolled. All patients received a clopidogrel loading dose of 600 mg followed by a daily dose of 75 mg. Blood samples from patients were obtained from the arterial sheath (6F) in the catheterization laboratory directly post PCI and at least 5 minutes after intravenous infusion of aspirin. All analyses were performed by trained laboratory technicians blinded to the results of other test and to the outcomes. The study is reported according to the STROBE (strengthening the reporting of observational studies in epidemiology) standards.

### Platelet Aggregometry

Whole blood aggregation was determined using Multiple Electrode Aggregometry (MEA) on a new generation impedance aggregometer (Multiplate Analyzer, Verum Diagnostica GmbH, Munich, Germany) directly after blood sampling at the Department of Clinical Pharmacology at the Medical University of Vienna. The system detects the electrical impedance change due to the adhesion and aggregation of platelets on two independent electrode-set surfaces in the test cuvette [25]. We used hirudin as anticoagulant and adenosine diphosphate (ADP) + prostaglandin E1 (PGE1) as agonists [26]. A 1:2 dilution of whole blood anticoagulated with hirudin and 0.9% NaCl was stirred at 37°C for 3 min in the test cuvettes, ADP: 6.4 µM and PGE1: 9.4 nM were added and the increase in electrical impedance was recorded continuously for 6 min [27]. The mean values of the 2 independent determinations are expressed as the area under the curve of the aggregation tracing (AUC = AU*min) and reported in U (10 AU*min = 1 U). Values >48U corresponded to HTPR [24].

### Genotyping

Genotyping was performed after inclusion of the last participant at the Institute of Molecular and Forensic Genetics, Collegium Medicum of the Nicolaus Copernicus University in Bydgoszcz, Poland. Genomic DNA was extracted from blood according to the standard procedures. CYP2C19*17 (CYP2C19_-806_C>T, rs12248560) was genotyped with a commercially available validated drug metabolism genotyping assay (TaqMan Drug Metabolism Genotyping Assay C_469857_10, Life Technologies, Carlsbad, California) with the ABI Prism Sequence Detector 7000 (Life Technologies) in accordance with manufacturer's instructions. CYP2C19*2 (CYP2C19_681_G>A; rs4244285) was genotyped with real-time allelic discrimination assay on an ABI Prism Sequence Detector 7000 (Life Technologies) according to standard procedures. Primers 5′- GATATGCAATAATTTTCCCACTATCATTG-3′ and 5′-GGTGTTCTTTTACTTTCTCCAAAATATCAC-3′ were used to amplify a sequence of the CYP2C19 gene containing the single nucleotide polymorphism 681G>A (rs4244285). The sequence of the G allele-specific probe was 5′-FAM-TTATTTCCCGGGAACC-3′ and the sequence of the A allele-specific probe was 5′-VIC-ATTATTTCCCAGGAACC-3′. SNPs in ABCB1 (rs1045542) and PON1 (rs662) were genotyped using commercial *TaqMan SNP Genotyping Assays* (assay IDs: rs1045642: C_7586657_20; rs662: C_2548962_20) on a *ViiA 7 Real-Time PCR System* (Life Technologies) following the manufacturer's instructions. After PCR, fluorescence yield for the two different dyes was measured and presented in a two-dimensional graph to obtain the allelic discrimination plot and identify individual genotypes. Correctness of genotyping was evaluated for randomly selected samples by direct sequencing of PCR products with the use of BigDye Terminator v. 3.1 sequencing kit and 3130xl Genetic Analyzer (Life Technologies). No discrepancies were observed between real-time discrimination and sequencing strategies.

### Study endpoint

The clinical endpoint was the composite of major adverse cardiac events (MACE: stent thrombosis: definite and probable, ACS and cardiac death) during a 12-month follow up. Stent thrombosis was defined according to the Academic Research Consortium criteria as the occurrence of an ACS with either angiographic or pathological confirmation of thrombosis [28]. Probable stent thrombosis was defined as any unexplained death within 30 days or target vessel myocardial infarction without angiographic confirmation of thrombosis or other identified culprit lesion [28].

### Statistical analysis

Based on a 22% rate of a composite of major adverse events in the group with high on treatment platelet reactivity (HTPR) compared to 8% in the group without HTPR [29], we calculated that 416 patients in the study would provide 99.9% power to detect significant differences (one sided alpha value of <0.05). Normal distribution was tested with the Kolmogorov Smirnov test. Data are expressed as mean, standard deviation (SD), 95% confidence intervals (CI) median or interquartile range. Statistical comparisons were performed with the t test, the Mann Whitney U test and the X^2^-test when applicable. Kaplan-Meier curves with the Breslow test were used for survival analyses. The Bonferroni correction was used for multiple comparisons. A multivariate Cox regression model was used to determinate independent predictors of MACE. The univariate logistic regression analysis was used to estimate variables responsible for HTPR and was a first step in the factor analysis. The effect of each variable on HTPR and MACE was tested using path analysis modelling, wherein the model fit was examined, as well as the significance of the direct and indirect effects (included variables: CYP2C19*2, CYP2C19*17, ABCB1 and PON1 carrier status, body mass index (BMI), diabetes mellitus, age, renal failure (creatinine clearance <60 mg/ml) and ACS at admission). Other gene environment interactions were tested in an exploratory factor analysis.

The following indicators were used to assess the goodness of fit of the models: Comparative Fit Index and Root Mean Square Error of Approximation. The maximum likelihood estimation method for structural equation modelling was used to test the conceptual model, examining the relationships among latent variables. The rationale for using the structural equation modelling instead of logistic regression in our paper is explained below. Logistic regression allows investigation of relationship between isolated independent variables and a single dependent variable. Therefore, regression analysis alone is inadequate when examining the interplay between the independent variables and several dependent variables. The structural equation modelling is more effective and more appropriate for analyzing complex models. The major difference between these two approaches is the mulicollinearity (when predictor variables are highly correlated). Whereas in a structural equation modelling, mulicollinearity is necessary, in a regression analysis mulicollinearity is problematic.

For the development of score predicting HTPR variables were selected by structural equation modeling and by forward and backward logistic regression. All statistical calculations were performed using commercially available statistical software (SPSS and AMOS, Version 21.0; Chicago).

## Results

### Patient Demographics

Patient demographics and co-medications are shown in Table 1. Two third of patients underwent non-emergent PCI and one third presented with an ACS on admission. Only five patients were lost to follow up during twelve months of follow-up.

**Table 1:** Patient demographics.

Patient Demographics	
	N = 416
Age (years)	64±12
Gender (male) n (%)	318 (76)
Risk factors/past medical history n (%)	
Body mass index (BMI; mean±SD)	28.1±5.5
Hypertension	352 (84)
Hyperlipidemia	318 (76)
Smoking	230 (55)
Family history of CAD	129 (31)
Diabetes mellitus	135 (32)
Prior PCI	197 (47)
Prior myocardial infarction	135 (31)
Peripheral arterial occlusive disease	54 (13)
Cerebrovascular disease	41(10)
Laboratory data (mean±SD)	
White blood cell count (WBC; ×10^9^/L)	7.9±2.6
Platelets (x10^9^/L)	224±71
C reactive protein (mg/dl)	1.3±1.2
Hemoglobin (g/dl)	13.3±1.9
Fibrinogen (mg/dl)	413±119
Creatinine (mg/dl)	1.3±0.9
Medication n (%)	
Aspirin	416 (100)
Clopidogrel	416 (100)
Proton pump Inhibitors (PPI)	317 (76)
β blockers	309 (74)
Angiotensin converting enzyme inhibitors (ACE)	219 (53)
Statins	303 (73)
Calcium channel blockers (CCB)	80 (19)
PCI data	
Elective PCI	274 (66)
PCI due to an acute coronary syndrome (ACS)	140 (34)
NSTE-ACS	67 (16)
STEMI	73 (18)
Number of stents per patient	1.7±1
Total stent length	31.8±21.7
CYP2C19*2 carrier status n (%)	126 (30)
CYP2C19*17 carrier status n (%)	165 (40)
ABCB1 carrier status n (%)	323 (77)
PON1 carrier status n (%)	210 (50)

Data are reported as Mean ± standard deviation (SD), n (number of patients) or percentages; CAD: coronary artery disease; PCI: percutaneous coronary intervention; NSTE-ACS: non ST- elevation acute coronary syndrome, STEMI: ST- elevation myocardial infarction. ABCB1: gene encoding transmembrane transporter P-glycoprotein; PON1: paroxonase 1.

### Genotype distribution

Thirty percent of patients were CYP2C19*2 carriers (27.6% heterozygote and 2.6% homozygote), 40% were CYP2C19*17 carriers (33.9% heterozygote and 5.8% homozygote), 78% had an ABCB1 C3435T genotype (55.6% heterozygote and 22.0% homozygote) and 50% were carriers of PON1 Q192R allele (44.2% heterozygote and 6.3% homozygote; Table 2).

**Table 2:** Univariate logistic regression for prediction of high on treatment platelet reactivity (HTPR).

Variable	HTPR N = 81 (20%)	no HTPR N = 321 (80%)	Regression coefficient	P value	OR	95% confidence intervals	
Age (years)	63±12	64±12	−0.014	0.225	0.986	0.964	1.009
Gender (male) n (%)	58 (72)	249 (78)	0.454	0.139	1.574	0.862	2.873
Risk factors/past medical history n (%)							
Body mass index (BMI; mean±SD)	29±5.8	28±5.2	0.036	0.141	1.036	0.988	1.086
Hypertension	66 (83)	275 (869	−0.210	0.559	0.810	0.400	1.641
Hyperlipidemia	60 (75)	248 (78)	−0.111	0.717	0.895	0.491	1.630
Smoking	46 (58)	174 (55)	0.128	0.650	1.137	0.653	1.979
Family history of CAD	21 (26)	105 (33)	−0.313	0.276	0.731	0.416	1.285
Diabetes mellitus	36 (44)	97 (30)	0.724	0.011	2.063	1.178	3.614
Peripheral arterial occlusive disease	9 (11)	45 (14)	−0.373	0.402	0.689	0.288	1.647
Cerebrovascular disease	7 (9)	34 (11)	0.114	0.812	1.121	0.437	2.875
Glomerular filtration rate (GFR: mean±SD)	78 (31)	85 (37)	0.006	0.137	1.006	0.998	1.014
Medications (%)							
Aspirin	81 (100)	321 (100)	−0.337	0.785	0.714	0.064	7.983
Clopidogrel	81 (100)	321 (100)	−41.805	0.999	0	0	0
Statins	63 (80)	257 (84)	−0.189	0.599	0.828	0.41	1.673
β blockers	65 (83)	256 (84)	−0.052	0.892	0.95	0.45	2.003
Proton pump inhibitors	68 (87)	246 (80)	0.357	0.354	1.429	0.672	3.04
Angiotensin converting enzyme inhibitors	54 (69)	210 (69)	−0.006	0.984	0.994	0.562	1.757
Calcium channel blockers	10 (13)	68 (22)	−0.719	0.06	0.487	0.231	1.03
PCI data							
PCI due to an acute coronary syndrome (ACS)	40 (50)	92 (29)	0.378	0.006	1.459	1.116	1.907
Number of stents per patient	1.86±1.27	1.69±0.98	0.204	0.085	1.226	0.972	1.547
Total stent length	33.7±24.7	31.1±20.9	0.005	0.36	1.005	0.994	1.016
Genetic data							
CYP2C19*17	24 (30)	135 (42)	−0.616	0.038	0.540	0.302	0.966
CYP2C19*2	33 (41)	90 (28)	0.477	0.070	1.611	0.929	2.796
ABCB1 C3435T	63 (77)	250 (77)	0.057	0.859	1.059	0.561	1.997
PON1 Q192R	40 (49)	160 (50)	−0.147	0.592	0.864	0.505	1.476

ACS: acute coronary syndrome; BMI: body mass index; GFR: glomerular filtration rate; CAD: coronary artery disease; CYP: cytochrome P450; ABCB1: gene encoding transmembrane transporter P-glycoprotein; PON1: paroxonase 1.

### Univariate influence of baseline characteristics and genetic polymorphisms on high on treatment platelet reactivity (HTPR)

Twenty percent of patients presented with a HTPR phenotype. Diabetes mellitus (OR: 2.1; 95%CI: 1.2–3.6; p = 0.011), ACS (OR: 1.5; 95%CI: 1.2–1.9; p = 0.006) and CYP2C19*17 genotype (OR: 0.54; 95%CI: 0.30-0-97; p = 0.038) emerged as HTPR predictors (Table 2). CYP2C19*2 genotype predicted HTPR only at 7% significance level (OR: 1.6; 95%CI: 0.93–2.80; p = 0.07; Table 2). No other variables differed between groups.

### Suggested path model explaining associations between genetic and clinical variables

**Figure 1:**
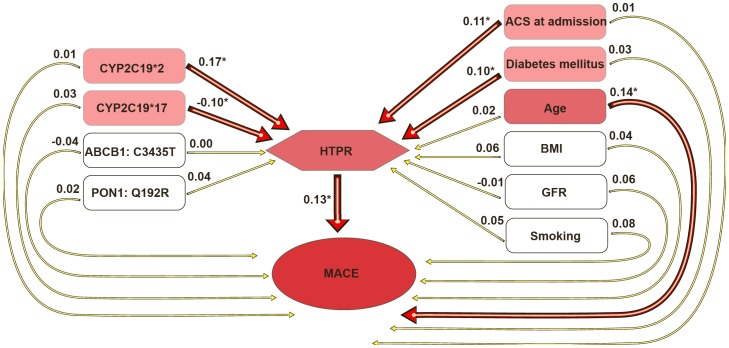
Path model of independent variables predicting high on treatment platelet reactivity (HTPR) and major adverse cardiac events (MACE: the composite of acute coronary syndrome, stent thrombosis and cardiac death). Paths from independent to dependent variables represent standardized estimates. *p<0.05; ACS: acute coronary syndrome; BMI: body mass index; GFR: glomerular filtration rate; CYP: cytochrome P450; ABCB1: gene encoding transmembrane transporter P-glycoprotein; PON1: paroxonase 1.

**Figure 2:**
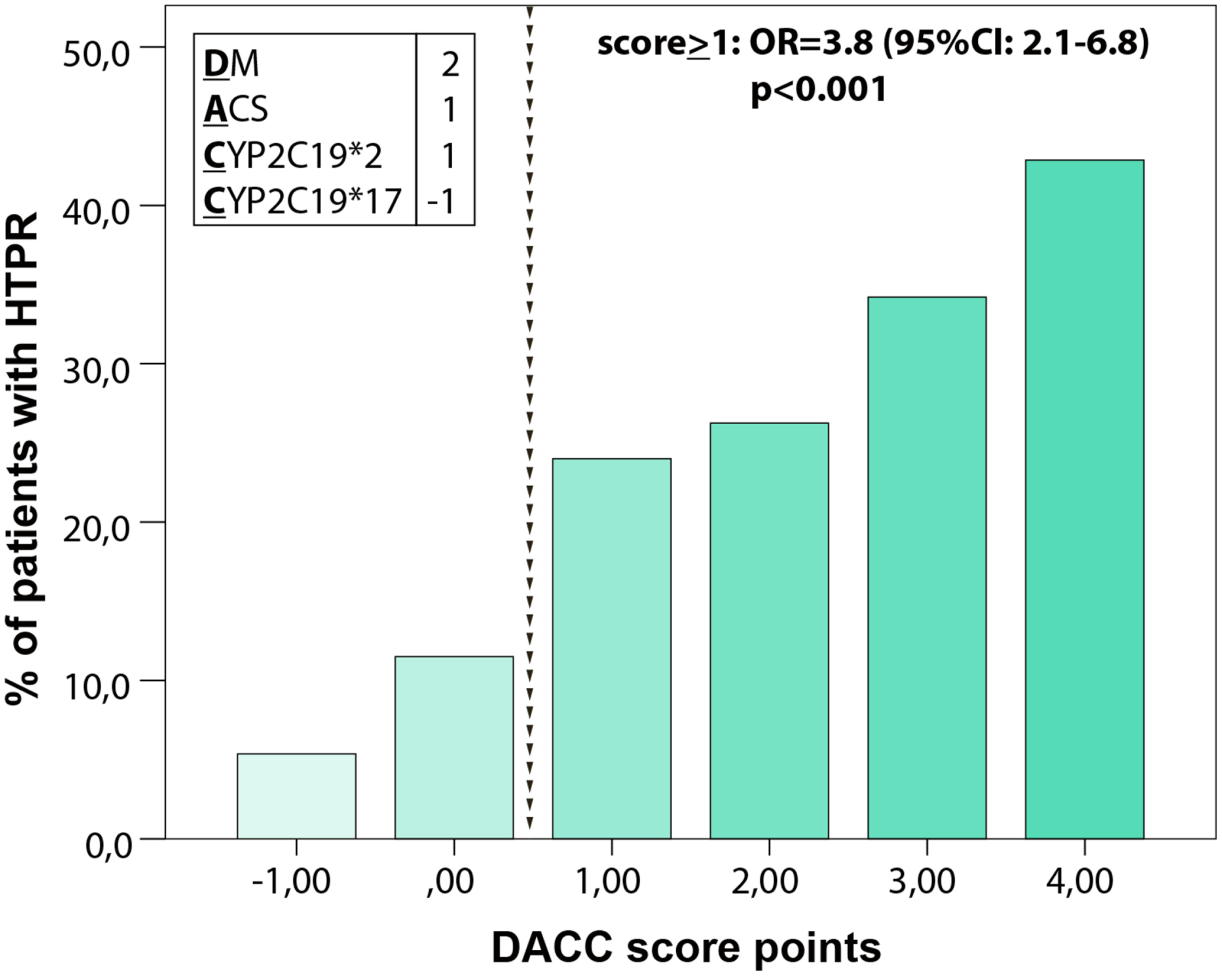
Incidence of increased % of patients with high on treatment platelet reactivity (HTPR) according to cumulative number of score variables. ACS: acute coronary syndrome; DM: diabetes mellitus; CYP: cytochrome P450.

Genetic and clinical variables were included into the model (Figure 1). The path model presented very good fit (Root Mean Square Error of Approximation = 0.000, Comparative Fit Index = 1.000). The paths from genetic polymorphisms of CYP2C19*2 and *17 (hetero or homozygote) as well as from clinical characteristics as ACS on admission and diabetes mellitus were independent predictors of HTPR (path coefficients: 0.17, -0.10, 0.11 and 0.10. respectively; p<0.05 for all; Figure 1; Table 2 and 3). In contrast, polymorphisms of ABCB1 or PON1 genes or other clinical characteristics were not depicted as independent predictors of HTPR in the model. From all included variables (genetic and clinical), only HTPR and age were independent predictors of MACE (path coefficient: 0.14 and 0.13, respectively; p<0.05; Figure 2; Table 2 and 3).

**Table 3:** Path modelling results.

dependent variable		path precursor	standardized estimate/path coefficient	standard error	P value
**HTPR**	<---	CYP2C19*2	0.165	2.814	0.001
	<---	CYP2C19*17	−0.103	2.643	0.035
	<---	ABCB1 C3435T	−0.010	3.092	0.838
	<---	PON1 Q192R	0.039	2.577	0.417
	<---	ACS at admission	0.109	1.305	0.025
	<---	Diabetes mellitus	0.096	2.755	0.048
	<---	BMI	0.062	0.244	0.207
	<---	GFR	−0.009	2.384	0.850
	<---	Smoking	0.047	2.612	0.333
	<---	Age	0.023	0.104	0.638
**MACE**	<---	CYP2C19*2	0.012	0.037	0.803
	<---	CYP2C19*17	0.034	0.034	0.497
	<---	ABCB1 C3435T	−0.040	0.040	0.418
	<---	PON1 Q192R	0.019	0.033	0.694
	<---	ACS at admission	0.014	0.036	0.770
	<---	Diabetes mellitus	0.033	0.017	0.508
	<---	BMI	0.041	0.003	0.409
	<---	GFR	0.061	0.031	0.224
	<---	Smoking	0.080	0.034	0.104
	<---	Age	0.141	0.001	0.004
	<---	HTPR	0.126	0.001	0.016

ACS: acute coronary syndrome; BMI: body mass index; GFR: glomerular filtration rate; CYP: cytochrome P450; ABCB1: gene encoding transmembrane transporter P-glycoprotein; PON1: paroxonase 1; HTPR: high on treatment platelet reactivity; MACE: major adverse cardiac events (MACE: the composite of acute coronary syndrome, stent thrombosis and cardiac death).

No other gene environment interactions were found.

### Development of a risk score

By using of the following four factors: diabetes mellitus, ACS, CYP2C19*2 and CYP2C19*17 a cumulative score was formed and applied on the total patient cohort to analyze its predictive value for HTPR. To account for the unequal influence of score variables, we allocated a weighing factor of -1 to 2 to each of the variables depending on the OR (-1 = OR<0; 1 = OR>1 but <2; 2 = OR>2). In detail, **D**iabetes was weighed by factor 2, **A**CS by factor 1, ***C*** YP2C19*2 by factor 1 and ***C*** YP2C19*17 by factor -1 (DACC score). Thus, a score ranging from -1 to 4 was developed. Hereby, we found an increasing incidence of HTPR by cumulative number of score variables (Figure 2). In logistic regression analysis the risk of having HTPR was calculated with an OR of 3.8 (95%CI: 3.1–6.8, p<0.001) for a score level of ≥1 compared with a score of <1 (Figure 2).

### Survival analysis

**Figure 3:**
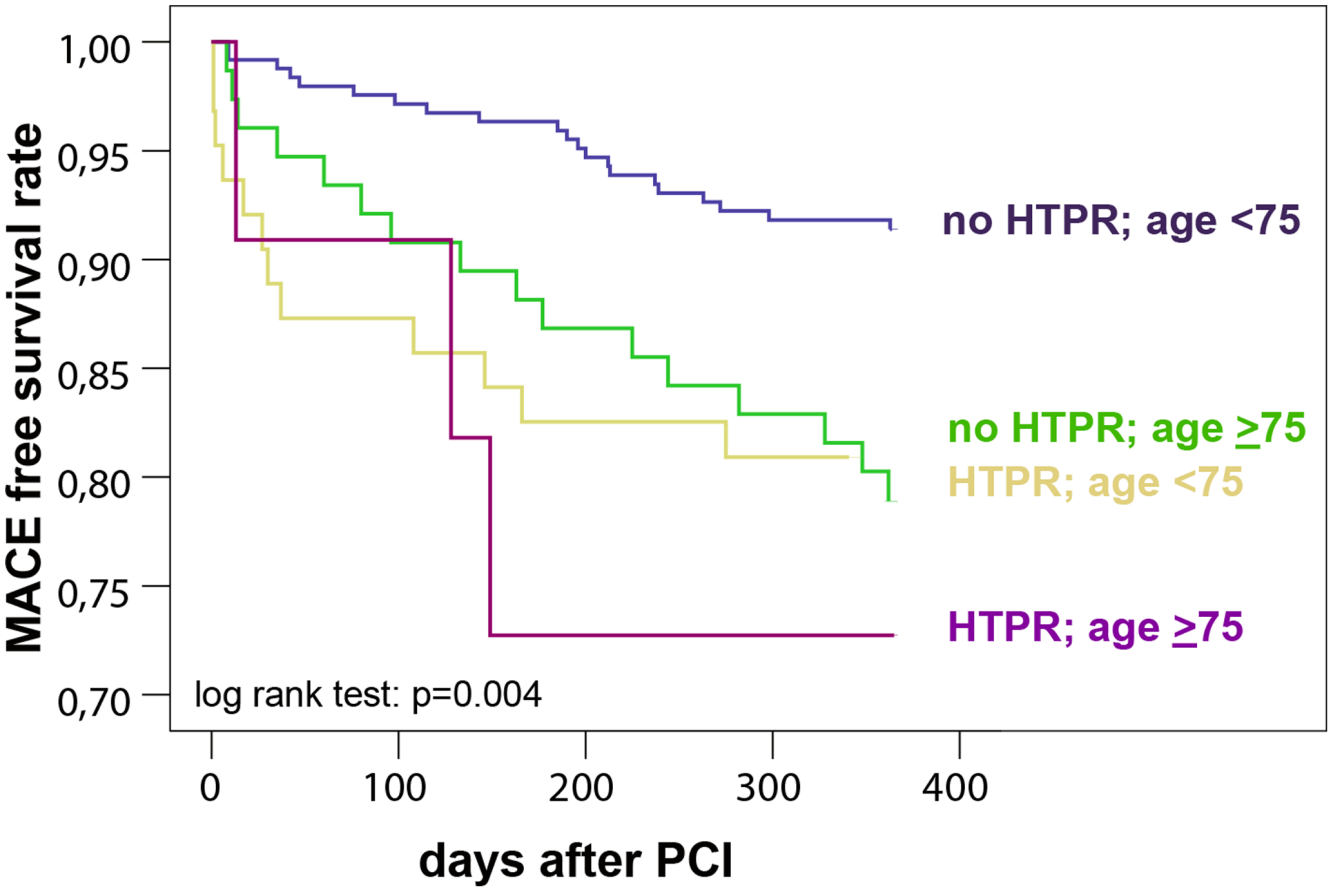
Survival analysis according to the high on treatment platelet reactivity (HTPR) and age. MACE: major adverse cardiac events: the composite of acute coronary syndrome, stent thrombosis and cardiac death; PCI: percutaneous coronary intervention.

The composite of major adverse cardiac events (MACE: stent thrombosis, ACS and cardiac death) occurred in 52 patients (12.5%). Cox multivariate adjusted model confirmed that HTPR and age independently predicted MACE: patients with HTPR were at 2-fold higher risk (95%CI: 1.1–3.6; p = 0.027; Table 4), whereas patients with ≥75 years of age had a 2.3-fold higher risk (95%CI: 1.3–4.4; p = 0.008; Table 4). A significant risk increase was observed after stratification of patients according to the HTPR and age: those with HTPR and age ≥75 years suffered the highest incidence of MACE: 27% during 12-month follow up (Figure 3). The lowest MACE rate occurred in patients younger than 75 years of age and without HTPR (9%; p = 0.004; Figure 3).

**Table 4:** Multiple Cox regression model for prediction major adverse cardiac events (MACE: the composite of acute coronary syndrome, stent thrombosis and cardiac death).

	Regression coefficient	P value	OR	95% confidence intervals	
HTPR	0.677	0.027	1.968	1.078	3.592
Age	0.845	0.008	2.38	1.248	4.345

HTPR: high on treatment platelet reactivity.

### Adapted path model

**Figure 4:**
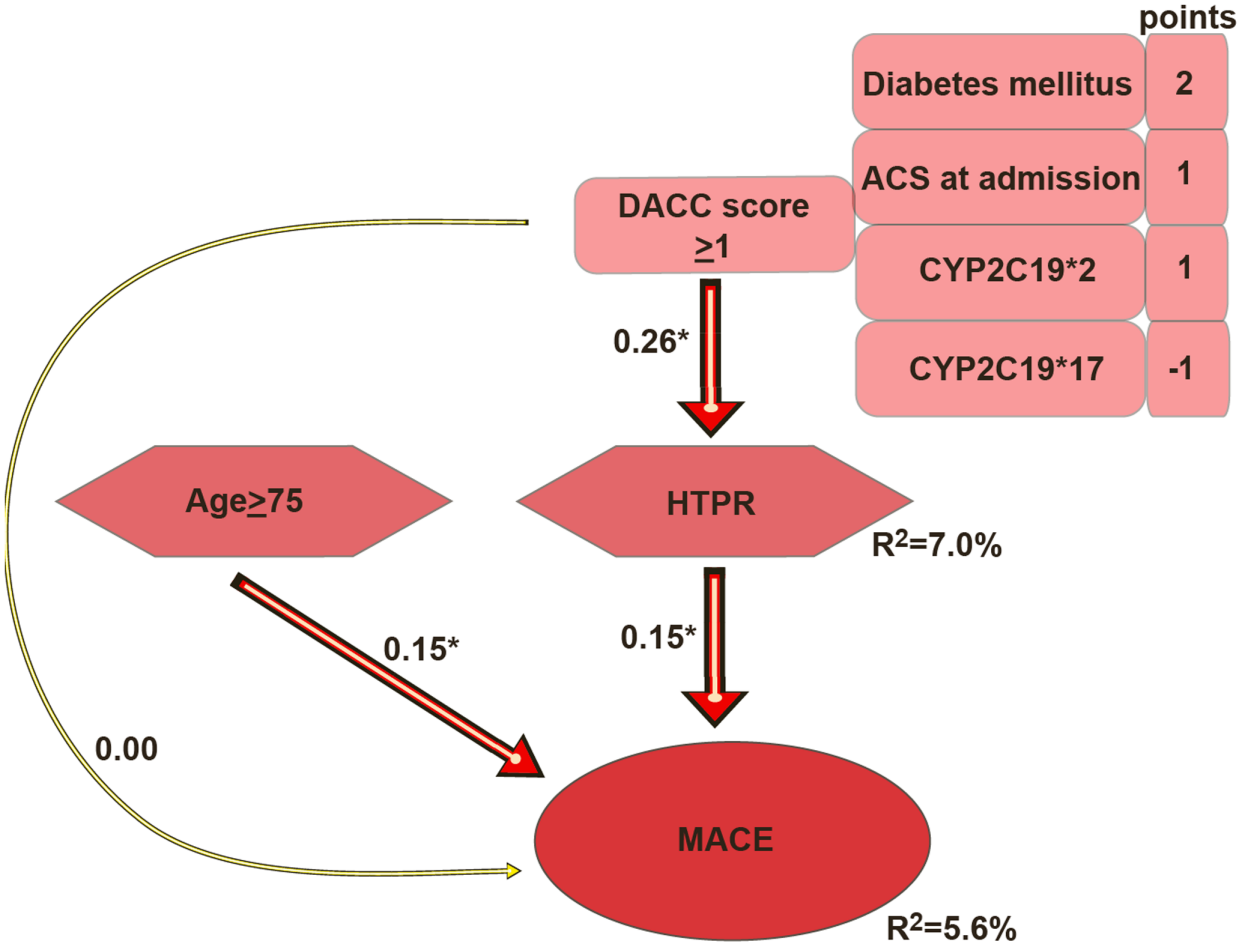
Adapted path model including the DACC score as an independent variable predicting high on treatment platelet reactivity (HTPR); Age and HTPR as independent predictors of major adverse cardiac events (MACE: the composite of acute coronary syndrome, stent thrombosis and cardiac death). Paths from independent to dependent variables represent standardized estimates. *p<0.05; ACS: acute coronary syndrome; CYP: cytochrome P450.

Based on the results of the DACC score and survival analyses, we adapted the previous path model. Whereas the DACC score explained 7.0% in the variance of HTPR (p<0.001; Figure 4), HTPR and age ≥75 years explained 5.6% in the variance of MACE p = 0.004, p = 0.003; respectively; Figure 4). Although the DACC score predicted HTPR, it did not predict MACE (Figure 4).

To verify the results of the path analysis, we performed a survival analysis looking at the event rates for each DACC score and there was no statistical difference. This confirms the results of the path analysis, which indicates that while DACC score predicts HTPR it does not predict MACE.

## Discussion

The central findings of this paper investigating a concurrent impact of clinical and genetic variables on HTPR and clinical outcome are as following:

- Two clinical characteristics (ACS and diabetes mellitus) and two genetic variants (CYP2C19*2 and CYP2C19*17) independently predicted HTPR but not MACE.
- By use of those four variables a score can be built, which allows estimating the probability of HTPR.
- While MACE was independently predicted by HTPR and age, the coexistence of HTPR and age ≥75 years emerged as the strongest predictor of MACE.

The findings of our study are interesting, since they pointed out for the first time the pathways of different variables leading to adverse outcomes. The first segment in the pathway leading to HTPR was built upon two genetic variants and two clinical variables. The CYP2C19*2 and *17 affect HTPR in different directions. As CYP2C19*2 reduces clopidogrel activation, it is a positive predictor of HTPR. In contrast, CYP2C19*17 which intensifies activation of clopidogrel, correlates negatively with HTPR. PON1 and ABCB1 polymorphism did not have an impact on HTPR. Therefore, our observation is in line with previous reports [30]–[33]. From several clinical variables reported to be associated with clopidogrel, only ACS and diabetes mellitus independently and positively predicted HTPR in this model. Both variables are factors known to be associated with HTPR [11], [34].

The suggested pathway examined with structural equation modelling might give a satisfactory explanation why many discrepancies exist in regard to whether or not genetic factors might predict clinical outcome. The association between CYP2C19*2 and adverse cardiovascular events postulated in some studies [12], [14]–[18], has not been confirmed in others [24], [35], [36]. Our model indicates that whereas platelet function testing identifies patients with HTPR, pharmacogenomic testing provides only a weak risk marker for HTPR. Platelet function testing provides therefore more comprehensive information than genotyping, as it reflects the influence of intrinsic (co-morbidities, drug-drug interactions) and genetic factors on the response to antiplatelet drugs. Nevertheless, in patients undergoing elective PCI (RAPID GENE study) or presenting with STEMI (RAPID STEMI study) pharmacogenomic testing with a subsequent use of prasugrel has been shown to eliminate HTPR [37], [38]. It is, however, still unknown whether pharmacogenomic approach will improve patient's outcome. Hopefully, undergoing trials as GIANT (NCT01134380) or TAILOR-PCI (NCT01134380) will deliver the missing answer to the above stated question. Importantly, it is still a matter of debate to define those patient cohorts, in whom platelet function testing or pharmacogenomics testing would be of clinical importance.

The second segment in our path model leading to MACE consisted of HTPR and age. Moreover, the coexistence of HTPR and age ≥75 years was a good risk stratifier for ischemic adverse events. The association between HTPR and adverse ischemic events is well characterized [2]–[8]. Higher age seems to be a universal clinical marker of risk. Age as a cofactor to HTPR, was less well described. Interestingly, one would presume that more pronounced platelet inhibition would be necessary in older patients. Surprisingly, this assumption could not be confirmed in the TRITON TIMI-38 study, showing that prasugrel was not superior to clopidogrel in older patient population but caused more bleeding events [39]. The latter aspect might be due to the fact that age has been identified as a baseline risk factor associated with both bleeding and ischemic events [40].

Accordingly, the combination of HTPR and age ≥75 years predicted MACE in 27% of cases, but explained only 5.6% of the variability in the occurrence of MACE during 1 year of follow-up. Nevertheless, it is unknown how our score compares with the known models of prediction of MACE based on traditional cardiovascular risk factors, as to our knowledge, the established scores did not report the R2 value.

Our model indicates that the two genetic variants CYP2C19*2 and *17 as well as the two clinical variables ACS and diabetes mellitus explain only 7% of the variability in platelet inhibition by clopidogrel. Similarly, previous reports showed that the CYP2C19*2 carrier status accounted only for 5–12% of the variability in the platelet response to clopidogrel [18], [41]. Thus, available data suggest that other variables like unknown genetic variants or other not identified factors contribute to this phenomenon.

Based on the results of the multivariate logistic regression and the structural equation modelling we developed a DACC score for prediction of HTPR. Interestingly, only four variables: two genetic variants and two clinical variables were necessary, to build the score. What was even more interesting, one point in the score was already satisfactory for prediction of HTPR. Three to four points in the DACC score predicted HTPR with a probability of 35–40%. This can be directly interpolated to the clinical practice: even without genetic testing it is probable by a factor 5 that a diabetic patient presenting with an ACS (3 score points) will have a HTPR. If this is the case, and if the patient is older than 75 years of age, the probability to develop MACE increases by factor 3 as compared to a younger patient without HTPR. Based on this example, the score might be useful in the clinical practice. Noteworthy, another score for prediction of HTPR has been already proposed. The weighted PREDICT score includes ACS, diabetes mellitus, left ventricular function, renal failure, age and CYP2C19*2 genotype, ranging a maximum of 165 points [42]. The disadvantage of the PREDICT score might be the limited availability of left ventricular function tests and a somehow challenging 2-step calculation algorithm with a requirement of a nomogram for estimation of HTPR probability. Nevertheless, both scores might offer a complementary information. Prospective studies would be required to test the usefulness of the scores in order to improve the management of antiplatelet agents and the net clinical outcome in the routine use.

Until now, only geno- and phenotyping but not scoring systems were used to personalize the antiplatelet therapy. Several studies have demonstrated that HTPR can be reduced with higher loading or maintenance doses of clopidogrel, or by switching to prasugrel or ticagrelor. Administration of a 150 mg maintenance dose of clopidogrel or up to four repeated loading doses of clopidogrel resulted in more intense inhibition of platelet aggregation in a major subset of patients but not in all [9], [43], [44]. Novel P2Y_12_ receptor inhibitors such as prasugrel or ticagrelor also achieved a stronger platelet inhibition in patients with HTPR under clopidogrel [9], [45], [46]. With regard to genotype-based personalized treatment, increased loading doses of clopidogrel up to 900 mg or maintenance doses up to 300 mg have been show to overcome clopidogrel non-responsiveness in heterozygous carriers of CYP2C19*2 allele but not in homozygous carriers [47], [48]. Although more potent platelet inhibitors prasugrel and ticagrelor became available in the ACS setting, our findings might still be important. Firstly, because clopidogrel is still the only authorized agent in patients undergoing elective PCI. Secondly, clopidogrel is widely used in ACS patients in some countries due to an economic impact since clopidogrel generics have entered into the market. Furthermore, recent studies in patients suffering from an ACS suggest that HTPR also occurs in patients treated with prasugrel or ticagrelor, especially in the early phase of treatment [49].

## Limitations

We are aware of the fact that the antiplatelet drug response is a multifactorial phenomenon, which cannot be solely explained by identified risk factors, because baseline differences in platelet aggregation are even observed in patients naive to antiplatelet treatment. As a further limitation, additional procedural factors during coronary intervention that might play an important role (e.g. type of lesion or procedure al time) or further genetic variants (e.g. ITGB3 encoding the integrin Beta3 of the GpIIb/IIIa receptor, P2Y_12_ receptor or insulin receptor substrate IRS-1) were not considered in our study. Moreover, due to the limited sample size, the study would not have enough power to include into the model and score the differentiation between homo- and heterozygotes of the CYP2C19*2 or 17* polymorphisms or to test the predictors of bleeding events. Most importantly, the absence of a validation cohort makes the generalizability of the DACC score difficult to predict.

## Conclusion

Our study suggests a pathway, which might explain the association between the genetic and clinical variables influencing the phenotype of response to clopidogrel. Furthermore, the proposed model also shows indirect and direct impact of several variables on clinical outcome: ACS, diabetes mellitus, CYP2C19*2 and CYP2C19*17 genetic variants independently predicted HTPR. In turn, age ≥75 years and HTPR were the strongest predictors of MACE. Further studies are needed to investigate the usefulness of our finding.
